# Comparative Analysis of Microalgae’s Physiological Responses to Fibrous and Layered Clay Minerals

**DOI:** 10.3390/biology14060647

**Published:** 2025-06-03

**Authors:** Zhongquan Jiang, Tianyi Wei, Sijia Wu, Zhongyang Wang, Zhonghua Zhao, Lu Zhang, Ying Ge, Zhen Li

**Affiliations:** 1 State Environmental Protection Key Laboratory of Environmental Health Impact Assessment of Emerging Contaminants, School of Environmental Science and Engineering, Shanghai Jiao Tong University, Shanghai 200240, China; zhongquanjiang@outlook.com (Z.J.); sardine0722@sjtu.edu.cn (T.W.); 2 College of Resources and Environmental Sciences, Nanjing Agricultural University, Nanjing 210095, China; 9201310523@stu.njau.edu.cn (S.W.); 9191310403@njau.edu.cn (Z.W.); lizhen@njau.edu.cn (Z.L.); 3 East China Sea Fisheries Research Institute, Chinese Academy of Fishery Sciences, Shanghai 200090, China; 4 State Key Laboratory of Lake Science and Environment, Nanjing Institute of Geography and Limnology, Chinese Academy of Sciences, Nanjing 210008, China; zhzhao@niglas.ac.cn

**Keywords:** clay minerals, microalgae, physiology, RNA-Seq, 3D-EEM

## Abstract

Microalgae interact with mineral particles in an aqueous environment, yet how clay minerals affect physiological processes in algal cells remains unexplored. In this study, we compared the effects of palygorskite (Pal) and montmorillonite (Mt), which respectively represent fibrous and layered clay minerals, on the physiological processes of *Chlamydomonas reinhardtii*. It was observed that *C. reinhardtii* responded differently to the treatments of Pal and Mt. The Pal particles bound tightly to and even inserted themselves into cells, resulting in a significant decrease in cell numbers from 27.35 to 21.02 × 10^7^ mL^−1^. However, Mt was only loosely attached to the cell surface. The photosynthesis in the algal cells was greatly inhibited by Pal, with the rETR_max_ significantly reduced from 103.80 to 56.67 μmol electrons m^−2^s^−1^ and the downregulation of IF2CP, psbH and OHP1, which are key genes involved in photosynthesis. In addition, Pal reduced the quantities of proteins and polysaccharides in extracellular polymeric substances (EPSs) and the P uptake by *C. reinhardtii* when the P level in the culture was 3.15 mg/L. However, no significant changes were found regarding the above EPS components or the amount of P in algal cells upon the addition of Mt. Together, the impacts of fibrous Pal on *C. reinhardtii* was more profound than those of layered Mt.

## Simple Summary

Microalgae are key to aquatic ecosystems, supporting food chains and nutrient cycles. Clay minerals, common in water, can either help or hinder algae—boosting growth by providing nutrients or harming them by blocking light. This study compared two clays, flat-layered montmorillonite and needle-like palygorskite, testing their impact on *Chlamydomonas reinhardtii*, a model green alga. We measured the algae’s growth, photosynthesis, cell structure, slimy coatings, and genetic responses. The results revealed that montmorillonite may support algae better due to its high surface area, while palygorskite’s sharp fibers could damage cells. These findings help explain how clays naturally influence algae, offering insights for managing harmful blooms or promoting useful algae in clean energy and water treatment.

## 1. Introduction

Microalgae form the foundation of aquatic food webs and play a critical role in global carbon and nutrient cycling, as well as the energy flow within ecosystems [1,2]. Their physiological processes, such as flocculation and nutrient consumption, are heavily influenced by environmental factors. Clay minerals are a ubiquitous component of aquatic and sedimentary environments, which exhibit complex interactions with microorganisms [3,4]. They can provide essential habitats and nutrients for microbial communities, while also adsorbing metabolic exudates and promoting the formation of biofilms [5,6,7].

Existing research indicates that clay minerals exert biphasic effects on microalgal physiology. On one hand, clay minerals help mitigate heavy metal stress via adsorption, act as nutrient carriers to enhance algal growth and form aggregates with algal cells to protect chlorophyll from degradation [8,9]. On the other hand, clay minerals could restrict motility and nutrient uptake and release toxic metal cations (e.g., Al^3^^+^, Fe^3^^+^) [10]. Moreover, previous studies have shown that the clay mineral montmorillonite could alter water transparency and light availability, thereby inhibiting microalgae photosynthesis by 30% [11]. Such a duality has led to clays being employed both as algal growth promoters and as algaecides, since they have been reported achieving a >90% removal efficiency for *Microcystis aeruginosa* via cell aggregation and sedimentation [12,13].

Despite their ecological relevance, key questions persist regarding how clay mineral structures govern interactions with microalgae. Montmorillonite (Mt) is a layered phyllosilicate accounting for over 70% of smectite clay deposits in many aquatic environments and extensively used in wastewater treatment [14]. Palygorskite (Pal) is a fibrous magnesium aluminosilicate with a 3D structure, which is equally ubiquitous in the sediment of rivers and lakes [15]. Although both minerals share the same chemical composition, their structures diverge fundamentally, since Mt has weak interlayer electrostatic bonds and cation hydration, while Pal features a hydrogen bond-stabilized channel. Their distinct structural characteristics make them highly representative within the realm of clay minerals. Mt and Pal are likely to exert divergent effects on microalgae due to their distinct morphologies [16,17]. High-surface-area Mt (600–800 m^2^/g) could impede or facilitate nutrients’ uptake by microalgae at high concentrations than fibrous Pal (100–200 m^2^/g) [18]. However, Pal’s needle-like structure can mechanically puncture cells, thus interfering with microalgal cell motility, yet no direct comparisons exist [19]. Besides, while phosphorus (P) is known to critically regulate algal growth and species like *Chlamydomonas reinhardtii* can accumulate P at 2–4% of dry cell weight [20], how clays modulate P uptake, extracellular polymeric substance (EPS) secretion and the subsequent impact on the overall phosphorus cycle remains unclear.

This study is meant to address these gaps by systematically comparing the effects of Mt and Pal on *C. reinhardtii*—a model green alga integral to biogeochemical cycles. We evaluate physiological responses, EPS production and cell morphology, and transcriptomic shifts of *C. reinhardtii* under exposure to Mt and Pal. By linking mineral structure to algal cells’ functional outcomes, this work aims to advance strategies for clay-enabled algal regulation in aquatic ecosystems.

## 2. Materials and Methods

### 2.1. Algal Incubation and Mineral Treatments

*C. reinhardtii* (CC-125) was purchased from the Chlamydomonas Resource Center, Department of Plant and Microbial Biology, University of Minnesota, United States. It was initially cultured in Tris-Acetate-Phosphate (TAP) medium at 25 ± 2 °C (light/dark 12/12 h, light intensity 2000 Lux) [21]. The algal strain was then purified by streaking it on an agar plate with TAP medium and forming algal colonies. A 0.5 cm × 0.5 cm algal sample was collected from the plate and then placed in a 100 mL liquid TAP medium prior to the mineral treatments.

The Mt and Pal samples were provided by The Clay Minerals Society (collection at Purdue University, West Lafayette, IN, USA). They were stored in a desiccator, and no chemicals or treatments were applied to them before the following experiments. Mt and Pal samples were added into a 100 mL TAP medium to obtain concentrations of 0 mg/L, 200 mg/L and 500 mg/L. The 200 mg/L concentration aligns with the range found in natural freshwater systems, as Mt concentrations can reach 200–250 mg/L in Yellow River water during high runoff in areas with nearby clay-rich geological formations [22]. The 500 mg/L was selected to explore the upper-limit stress responses of the microalgae. The chosen concentrations for Pal were set to match those of Mt. For the mineral exposure, a 1 mL suspension of *C. reinhardtii* (in the logarithmic phase of growth) was incubated in the above mineral–TAP composite. The mineral treatments were denoted as Mt_0_, Mt_200_, Mt_500_, Pal_0_, Pal_200_ and Pal_500_, respectively. All the samples were cultured by shaking them three times every day during a four-day incubation. Additionally, in order to explore the ability for P uptake by *C. reinhardtii* under mineral treatments, we set two P concentrations, 3.15 mg/L and 31.5 mg/L, respectively in the algal culture. For other analyses, the P concentration in the culture medium was 3.15 mg/L. Three replicates were prepared for each treatment.

### 2.2. Photosynthesis Measurements

After four days of incubation, photosynthetic parameters Fv/Fm (maximum quantum yield) and rETR_max_ (maximum relative electron transport rate) were measured using a multiple excitation wavelength phytoplankton photosynthesis analyzer (Phyto-PAM-II Modular Version, Walz, Effeltrich, Germany). Cell density was determined by measuring optical density at 680 nm (OD_680_) with a plate reader (SpectraMax i3X, San Jose, CA, USA) and converting the values to cell counts using a pre-calibrated standard curve [23].

An amount of 4 mL of algal culture was centrifuged at 5000 rpm for 5 min, and the supernatant was discarded. The cell pellet was resuspended in an equal volume of methanol and incubated at 4 °C in the dark for 24 h to facilitate the extraction of pigment. Following extraction, the suspension was centrifuged again, and the absorbance of the supernatant was measured at 665 nm for chlorophyll a, 649 nm for chlorophyll b and 470 nm for carotenoids using a UV–Vis spectrophotometer.

### 2.3. Respiration Rate Assay

For respiration rate analysis, sterilized 320 mL saline bottles containing 100 mL of liquid TAP medium were inoculated with 1 mL of logarithmic-phase algal culture and specified mineral concentrations under aseptic conditions. Cultures were incubated for 96 h at 25 °C with 120 rpm shaking under a 12 h light/dark cycle (2000 Lux). Post-incubation, seals were replaced with sterile rubber stoppers, and headspaces were flushed with standard air for 3 min to ensure gas equilibration. Dark respiration was measured over 1 h in a temperature-controlled environment. Headspace gas samples (10 mL) were analyzed for CO_2_ using an Agilent GC-7890B with a Haysep Q column (2 m × 3 mm, 80/100 mesh) and thermal conductivity detector (TCD), while cell density was determined via OD_680_ measurements. These steps ensure the accurate quantification of respiration rates by separating metabolic CO_2_ production from photosynthetic activity, with GC parameters standardized for reproducibility.

### 2.4. Phosphorus Accumulation Analysis

Algal samples were centrifuged at 5000 rpm for 5 min, and the supernatant was filtered through a 0.45 μm membrane. Dissolved phosphorus in the filtrate was quantified to assess total P depletion from the medium, including P associated with cells or minerals and intracellular accumulation, using inductively coupled plasma mass spectrometry (ICP-MS, PerkinElmer NexION 2000, Waltham, MA, USA).

### 2.5. Fluorescence Spectroscopy of EPS

A fluorescence analysis of dissolved extracellular polymeric substances (EPSs) was performed using a fluorescence spectrophotometer (SHIMADZU RF-6000, Kyoto, Japan). Excitation (Ex) and emission (Em) wavelengths were scanned from 200 to 500 nm in 10 nm increments at a speed of 6000 nm/min, with a spectral bandwidth of 3 nm. For intracellular pigment extraction, the pelleted cells were resuspended in an equal volume of methanol, incubated at 4 °C for 24 h, and the absorbance of chlorophyll a (Chl a), chlorophyll b (Chl b) and carotenoids was measured using the plate reader [24].

### 2.6. Intra/Extracellular Protein and Polysaccharide Assays

To separate intra/extracellular components, the culture was directly filtered through a 0.45 μm PES membrane to collect soluble proteins and polysaccharides. Cells retained on the membrane were resuspended, heated at 50 °C for 2 h and sonicated to disrupt the cells, releasing total proteins and polysaccharides. The disrupted cell suspension was re-filtered through a PES membrane to separate soluble and cell surface-bound components.

Cell surface-bound proteins/polysaccharides were calculated as the difference between Ps+c and Ps. Intracellular proteins/polysaccharides (Pi) were determined as the difference between Pt and Ps+c. Protein content was measured using the bicinchoninic acid (BCA) method, and polysaccharides were quantified via the phenol–sulfuric acid digestion method [21].

### 2.7. Functional Groups and Cell Morphology

The algal sample was centrifuged and stored in a refrigerator for freeze-drying. Then, the functional groups were analyzed by ATR-IR (Nicolet iS5 FTIR, Thermo Fisher Scientific, Waltham, MA, USA) [15]. The centrifuged cells were fixed with ethanol and freeze-dried with acetone. The samples were stored for SEM analysis using Zeiss-Supra55 (Carl Zeiss Microscopy, Oberkochen, Germany) [25]. For TEM (Tecnai G2 F20S-TWIN, Eindhoven, The Netherlands + AZtec 6.2 X-Max 80T) observation, the samples were first fixed with glutaraldehyde and then stored at 4 °C. Finally, ultrathin (60–80 nm) was cut by a diamond knife for TEM analysis [26].

### 2.8. Transcriptomic Analysis

The transcriptome analyses were performed at the 200 mg/L mineral treatments for observation. After 96 h of cultivation, algal samples from each treatment were centrifuged at 8000 g for 10 min. Pellets were washed with sterile PBS buffer, and the supernatant was discarded. Total RNA was extracted from *Chlamydomonas* cells using a standard protocol, followed by quality assessment to ensure integrity for downstream analysis. Transcriptomic libraries were constructed using the NEB#7530 Kit (#E7530, New England Biolabs, Ipswich, MA, USA) according to the manufacturer’s instructions. Library quality was evaluated using the DNA 1000 Assay Kit (Agilent Technologies, Santa Clara, CA, USA, 5067-1504), which detects fragment sizes (25–1000 bp) and concentrations (0.1–50 ng/µL). Transcript expression levels were calculated using the TPM (transcripts per million reads) method, with gene abundances quantified by RSEM (http://deweylab.biostat.wisc.edu/rsem/ (accessed on 6 February 2022)). DEGs between samples were identified using DESeq2/DEGseq/EdgeR with thresholds: Q value ≤ 0.05, |log_2_FC| > 1, and Q value ≤ 0.001 (for DEGseq). GO and KEGG pathway analyses were performed using Goatools (https://github.com/tanghaibao/Goatools (accessed on 7 February 2022)) and KOBAS (http://kobas.cbi.pku.edu.cn/home.do (accessed on 7 February 2022)), with significance defined as Bonferroni-corrected *p* ≤ 0.05.

### 2.9. Data Analysis

All the data were analyzed by R project (version 4.1.0) and SPSS Statistics software (IBM version 17.0) with a significance level of *p* < 0.05, as well as Origin Pro 2025 being used for graph preparation. The significant differences between the control and treatment groups were determined using a one-way analysis of variance (ANOVA, Duncan). The data in the figures was all presented as the mean value with standard deviation (n = 3).

## 3. Results

### 3.1. Photosynthesis Parameters

**Figure 1:**
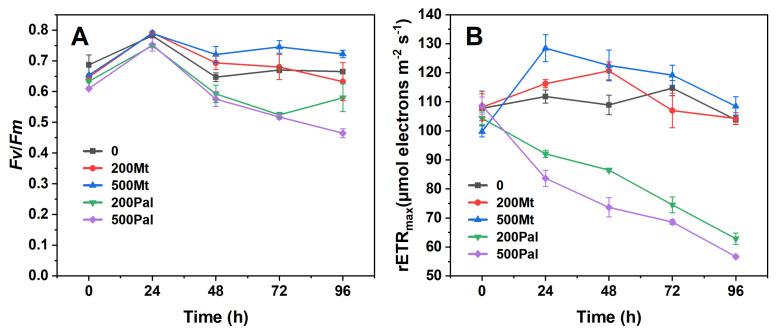
The effects of montmorillonite (Mt) and palygorskite (Pal) on (**A**) the maximum quantum yields of the photosystem II reaction center and (**B**) electron transport rate. The vertical bar represents the standard deviation of triplicate samples.

Under the treatments of Mt_200_ and Mt_500_, there was no significant change in Fv/Fm compared with the control group (Figure 1A). However, the value decreased significantly under Pal’s addition. When the alga was incubated with Pal_200_ and Pal_500_ for 72 h, the Fv/Fm data decreased from 0.6 to 0.5 (Figure 1A).

The rETR_max_ did not vary significantly upon Mt’s addition (Figure 1B), although it increased at 24 h and decreased afterwards. After 24 h, the rETR_max_ under Mt_500_ reached were 128.5 μmol electrons m^−^^2^s^−^^1^, and they remained higher than the control group. In contrast, Pal’s addition dramatically reduced the rETR_max_ values from about 105 to 62.9 μmol electrons m^−^^2^s^−^^1^ at Pal_200_ and 56.7 μmol electrons m^−^^2^s^−^^1^ at Pal_500_.

### 3.2. P Uptake, Cell Number and CO_2_ Emission Rate

**Figure 2:**
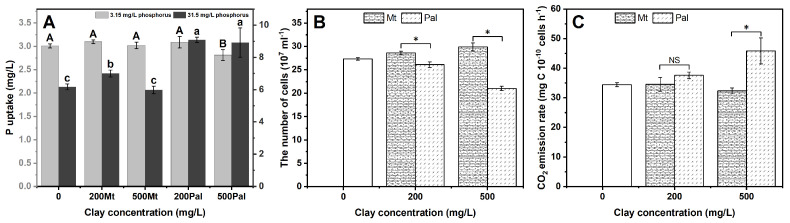
(**A**) P uptake of *Chlamydomonas reinhardtii* cultured at 3.15 mg/L and 31.5 mg/L P concentrations. (**B**) The number of counts (per 10^7^ cells) of *C. reinhardtii* after four days’ incubation. * *p* < 0.05. (**C**) CO_2_ emission (per 10^10^ cells) of *C. reinhardtii*. The error bars represent the standard deviation based on three parallel experiments. * *p* < 0.05; NS, not significant. Different uppercase letters indicate significant (*p* < 0.05) differences among various mineral concentrations at 3.15 mg/L P concentrations while different lowercase letters indicate significant (*p* < 0.05) differences at 31.5 mg/L P concentrations.

When the P supply was 3.15 mg/L, the P uptake declined from 3.0 to 2.8 mg/L at Pal_500_ (Figure 2A). When the P concentration in the medium was 31.5 mg/L, the P uptake increased significantly under Pal’s addition. However, the increase in P uptake was much lower under Mt’s addition (Figure 2A).

As for the growth in *C. reinhardtii*, Mt and Pal had contrasting effects. Mt_200_ and Mt_500_ respectively increased the cell number from 27.4 to 28.6 and 29.9 × 10^7^ cells/mL (Figure 2B). However, the number of cells significantly decreased to 26.1 and 21.0 × 10^7^ cells/mL treated by Pal_200_ and Pal_500_, respectively.

At Mt_200_, the respiration showed no evident fluctuation. When Mt was elevated to 500 mg/L, the CO_2_ emission rate slightly decreased from 35.0 to 32.3 mg C 10^−10^ cells h^−1^ (Figure 2C). Nevertheless, the CO_2_ emission rate showed a persistent increasing trend from Pal_0_ to Pal_500_, reaching 46.0 mg C 10^−10^ cells h^−1^.

### 3.3. Three-Dimensional Excitation–Emission Matrix Fluorescence Spectra

Fluorescence spectroscopy revealed distinct responses of extracellular polymeric substances (EPSs) to montmorillonite (Mt) and attapulgite (Pal) additions, categorized by three fluorescent components: tryptophan-like (C1), tyrosine-like (C2) and humic-like (C3/C4) substances.

**Figure 3:**
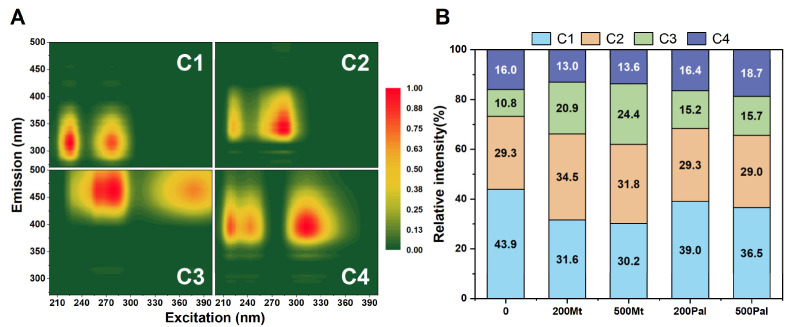
Three-dimensional excitation–emission matrix fluorescence spectra of the supernatant under different treatments. (**A**) The four fluorescent components (**C1**–**C4**) identified by PARAFAC analysis; (**B**): relative abundances of fluorescent components under different treatments.

C1 was characterized by peaks at 225/325 nm (dominant) and 225/290 nm (secondary), which exhibited significant intensity reductions under both Mt and Pal treatments. Mt_200_ and Mt_500_ decreased C1 to 31.6% and 30.2% of the control value, respectively, while Pal_200_ and Pal_500_ reduced it to 39.0% and 36.5% (Figure 3). In contrast, C2 (peak at 280/340 nm) showed an increased intensity by 34.5% and 31.8%, respectively, under Mt_200_ and Mt_500_ treatments, but no significant change was observed with Pal additions (Figure 3B).

Humic-like components C3 (located at 280/475 nm) and C4 (located at 290/460 nm) exhibited a weaker fluorescence compared to C1 and C2, with minor intensity fluctuations under mineral exposure. C3 showed a reduction of 10–15% for Mt and negligible changes for Pal, while C4 showed an increase for both Mt and Pal (Figure 3B).

### 3.4. Polysaccharides, Proteins, Chlorophyll and Carotenoids

**Figure 4:**
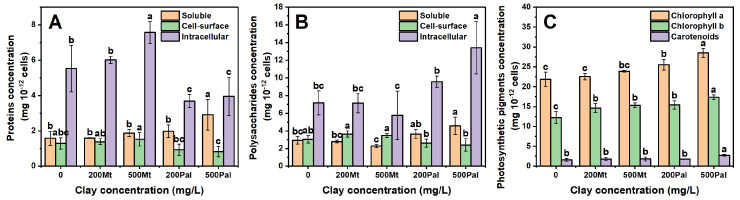
The content of proteins (**A**), polysaccharides (**B**) and photosynthetic pigments (**C**) after four days of incubation. Different lower case letters indicate significant (*p* < 0.05) differences among various mineral treatments.

Compared to the control group, Mt treatments did not cause significant changes in the amounts of soluble and cell surface proteins as shown in Figure 4A. However, the content of intracellular protein increased with rising Mt concentrations. The intracellular protein content reached 6.0 mg 10^−12^ cells at Mt_200_ and increased to 7.6 mg 10^−12^ cells at Mt_500_.

The amount of soluble proteins increased after the addition of Pal. For Pal_200_, the soluble protein content rose from 1.6 mg 10^−12^ cells to 2.0 mg 10^−12^ cells. For Pal_500_, it increased to 3.0 mg 10^−12^ cells (Figure 4A). Regarding intracellular protein, its content decreased significantly from 5.5 mg 10^−12^ cells to 3.7 mg 10^−12^ cells upon adding Pal_200_, while it increased slightly to 4.0 mg 10^−12^ cells with Pal_500_.

The content of soluble and intracellular polysaccharides decreased with the elevation in Mt, i.e., from 2.9 to 2.3 mg 10^−12^ cells and from 7.2 to 5.8 mg 10^−12^ cells, respectively (Figure 4B). In contrast, these polysaccharides increased with the elevation in Pal. Upon Pal_500_, the intracellular polysaccharides increased remarkably from 7.2 to 13.4 mg 10^−12^ cells. The cell surface polysaccharides content hence showed an increasing trend under Pal’s addition.

The changes in chlorophyll a, chlorophyll b and carotenoids in cells showed an upward trend under the addition of Mt and Pal (Figure 4C). Following the addition of Mt_500_, chlorophyll a increased from 21.9 to 23.9 mg 10^−12^ cells, whereas this value increased to 28.5 mg 10^−12^ cells at Pal_500_. In addition, upon the 500 mg/L Mt and Pal treatments, the content of chlorophyll b rose from 12.2 to 15.3 mg 10^−12^ cells and to 17.4 mg 10^−12^ cells, respectively. The carotenoid content did not change significantly at Mt_500_, but it increased from 1.6 to 2.7 mg 10^−12^ cells at Pal_500_ (Figure 4C).

### 3.5. Functional Groups on the Cell Surface

**Figure 5:**
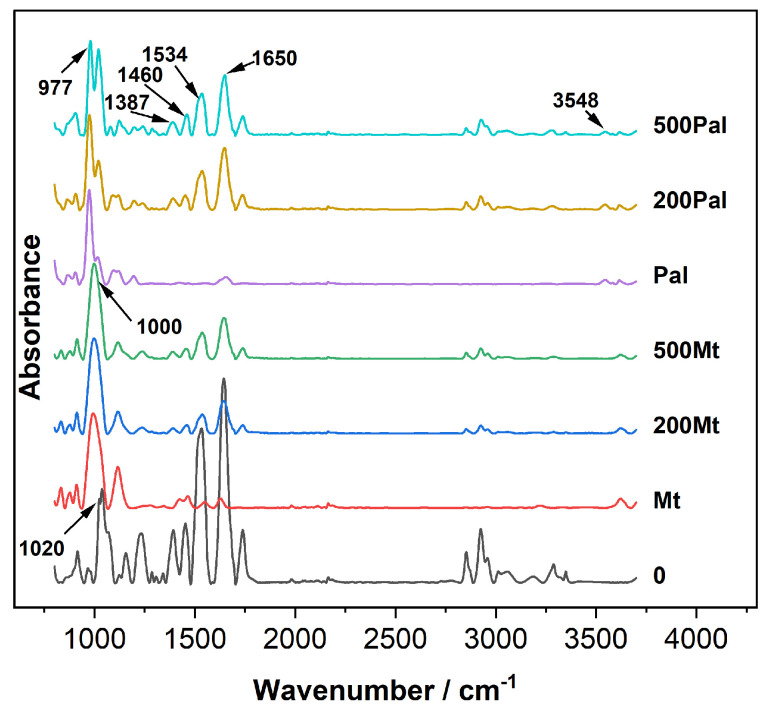
ATR−IR spectra of the *C. reinhardtii* cells incubated under 200, 500 Mt/Pal.

Attenuated total reflection–infrared (ATR-IR) spectroscopy was employed to investigate the functional groups present on the cell surface. The ATR-IR spectra presented peaks at 977 and 1000 cm^−1^, which were attributed to Si-O vibrations (Figure 5). A distinct peak at 1020 cm^−1^ was indicative of Si-O-Si vibrations. Notably, upon the addition of Mt and Pal, this Si-O-Si vibration peak vanished (Figure 5). This disappearance suggests a significant interaction between the silicate-containing minerals and the cell surface components, potentially involving the disruption or modification of Si-O-Si bonds.

The peaks at 1387 cm^−1^ and 1460 cm^−1^ corresponded to -COOH and P-O-H groups of polysaccharides, respectively. These functional groups play vital roles in various cellular processes, such as intercellular recognition and adhesion. The bands at 1534 and 1650 cm^−1^ were assigned to N-H and C=O groups in proteins. After the addition of Mt and Pal, the intensities of these peaks declined. This decrease in intensity signified a reduction in the content of amide groups, which is characteristic of proteins.

In addition, the peak at 3548 cm^−1^ was associated with the stretching vibration of -OH groups. Additionally, the overall peak intensities in the regions of 1500–1600 cm^−1^ and 2900–3500 cm^−1^ were weakened. This weakening was likely a result of the binding of these functional groups with the clay particles (Mt and Pal).

### 3.6. Cell Morphology

**Figure 6:**
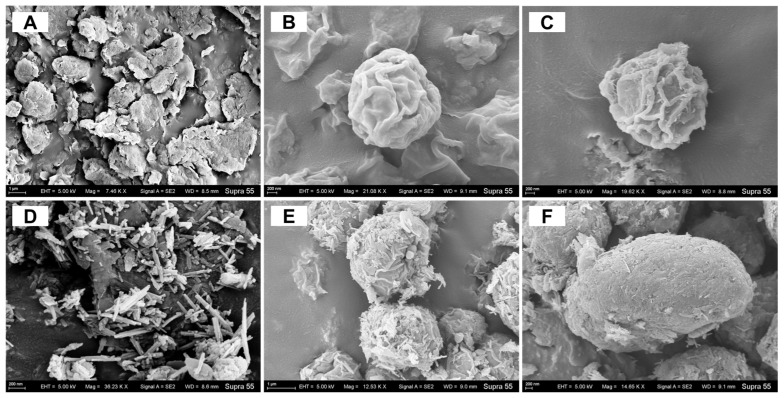
(**A**,**D**) SEM images of Mt (**A**) and Pal (**D**). (**B**,**C**,**E**,**F**): SEM imaging on the algae cells under 200 (**B**,**E**), 500 (**C**,**F**) Mt/Pal.

SEM images showed that the algal cells only weakly interacted with 200 mg/L Mt (Figure 6A,B). At Mt_500_, Mt particles were more tightly adsorbed by the cells (Figure 6C). In addition, abundant fibrous Pal particles adhered to the cell surface at Pal_200_ (Figure 6D,E). With the gradual elevation in Pal concentration, the density of mineral particles on the cell surface gradually increased. At Pal_500_, the cells were almost wrapped in Pal (Figure 6F), suggesting a strong binding between the Pal and algal cells.

**Figure 7:**
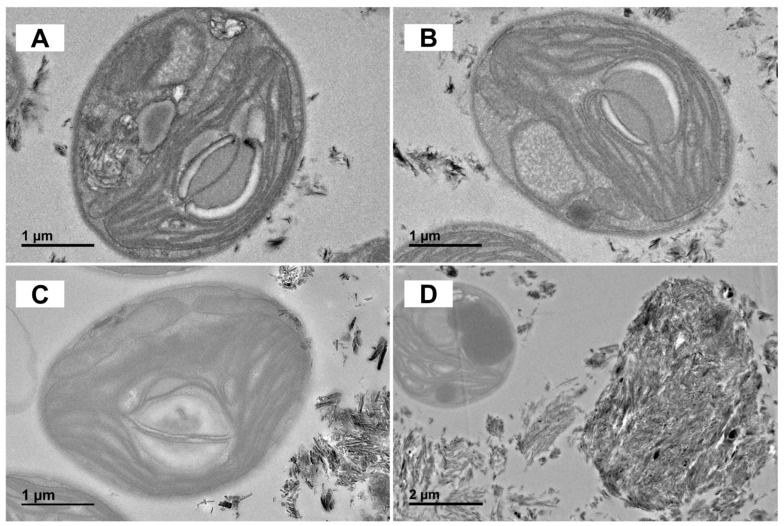
TEM images of *C. reinhardtii* cells incubated with 200 (**A**), 500 (**B**) mg/L Mt and 200 (**C**), 500 (**D**) mg/L Pal of the medium in all the treatments.

TEM observations showed that Mt crystals were adsorbed onto the cells at Mt_200_ (Figure 7A). With the increase in Mt addition, more Mt particles were observed on the cell surface (Figure 7B). In addition, there were only scarce mineral particles around the cell wall at Pal_200_, which appeared as separated mineral fibers (Figure 7C). The Pal particles even inserted themselves into the cell wall due to their fibrous shape. Compared with Mt, cell lysis could be observed, with large amounts of Pal adsorbed on the cell surface at Pal_500_ (Figure 7D).

### 3.7. Annotations of Differentially Expressed Genes (DEGs)

**Figure 8:**
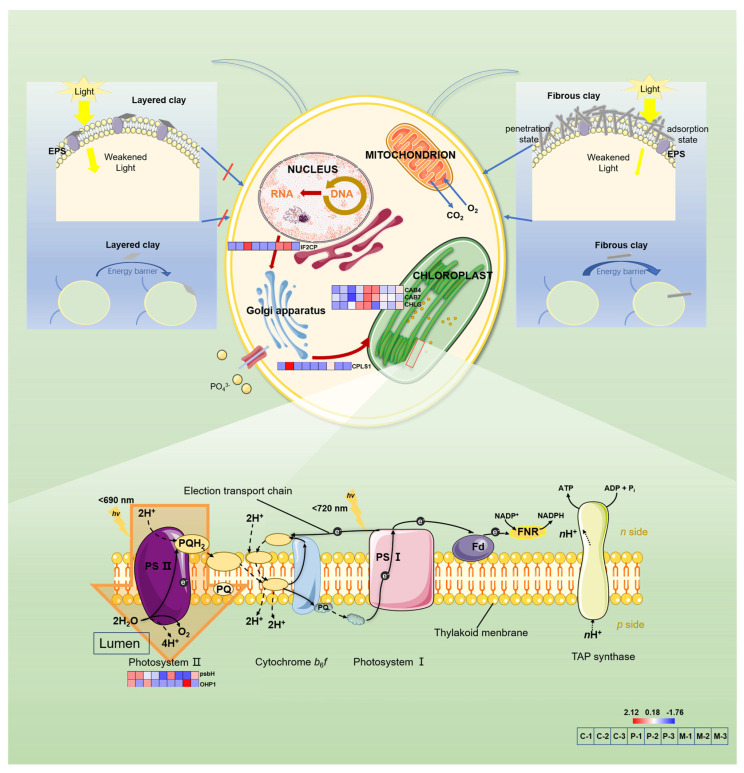
Main metabolic pathway network related to differentially expressed proteins (DEPs). The color bar indicates the degree of up–down adjustment of DEPs (C for CK; P for Pal (200 mg/L); M for Mt (200 mg/L)). IF2CP, CPLS1 genes: related to the transformation of precursor substances into chloroplasts; CAB4, CAB7 and CHLG genes: related to chlorophyll synthesis; SAMC1 and odhA genes: related to cellular respiration; psbH and OHP1 genes: related to Photosystem II synthesis.

Compared with the control, differentially expressed genes (DEGs) were identified in the 200 mg/L Mt and Pal treatments. Since photosynthesis is an important physiological process in algae, we focused on those significantly different genes involved in photosynthesis (Figure 8). IF2CP and CPLS1 genes are related to chloroplast synthesis based on the KEGG. After the addition of Pal, these genes were downregulated, which inhibited the chloroplast precursor material’s conversion to chloroplast. CAB4, CAB7 and CHLG genes regulate chlorophyll synthesis. These genes were significantly upregulated compared with the addition of 200 mg/L Mt. In addition, psbH and OHP1 genes related to PSII synthesis were significantly downregulated at Pal_200_. In addition, odhA and SAMC1 genes related to cellular respiration were upregulated at Pal_200_. However, the change was not evident in the gene expressions when Mt was added.

## 4. Discussion

The interactions between clay minerals and microorganisms have significant environmental implications within the biogeochemical cycle. For instance, microbial activities could modify minerals’ composition and enrich potassium (K), thereby creating a substantial K sink that contributes to balancing the imbalanced K budget in the ocean [27]. Furthermore, the potential of algae–mineral interactions in the remediation of heavy metal pollution, attributable to the substantial surface area to volume ratio of algae–mineral composites, has been suggested [28].

### 4.1. Differential Effects of Mt and Pal on Algal Photosynthesis

In this study, Pal exerted a significant inhibition on algal photosynthesis, whereas Mt showed a stimulation of mild growth. Pal’s interwoven fibrous particles tightly bound to the cell surface and even penetrated algal cells, inducing a dense aggregation on the cell surface (Figure 6E,F and Figure 7C,D). This physically obstructed light absorption, thus reducing photosynthetic parameters such as Fv/Fm and rETR_max_ (Figure 1). Fv/Fm reflects the quantum efficiency of photosystem II (PSII) by measuring the reduction state of the primary electron acceptor (Q_A_). Therefore, a decline indicates impaired PSII functionality in capturing light energy, thereby disrupting photosynthetic electron transport from PSII to photosystem I (PSI) [29]. Similarly, rETR_max_ serves as an indicator of the maximum capacity of the electron transport chain, with decreases reflecting reduced efficiency in electron flow through the algal thylakoid membrane [30].

As indicated by these two parameters, the photosynthesis of *C. reinhardtii* was severely inhibited by Pal particles. To compensate, chlorophyll content increased (Figure 4C), and light-harvesting genes (CAB4, CAB7, CHLG) were upregulated, which was consistent with the previous literature [31,32,33,34]. In contrast to Pal, Mt induced no significant alterations in algal photosynthesis (Figure 1). Although Mt’s particles were adsorbed onto the cell surface and partially obstructed light penetration, its layered structure allowed energy transfer through the mineral layer via light scattering [35]. This structural property of Mt minimized interference with light absorption and electron transport, explaining why photosynthetic parameters remained unimpaired under Mt treatments.

### 4.2. Phosphorus Uptake and Metabolic Pathway Responses

The presence of Pal was found to induce a 10% reduction in phosphorus uptake at 3.15 mg/L P (Figure 2A), disrupting metabolite synthesis and ATP-dependent processes critical for algal photosynthesis and growth [36]. Specifically, limited P availability constrained the synthesis of sugar phosphates and impaired photophosphorylation, as evidenced by the downregulation of IF2CP (translation initiation factor) and CPLS1 (chloroplast RNA splicing factor) genes (Figure 8). These effects cascaded into reduced Rubisco activity and Calvin cycle suppression, ultimately lowering photosynthetic efficiency [37]. Consequently, cell density decreased by 7% under Pal_200_ and 30% under Pal_500_ (Figure 2B), reflecting impaired metabolic support for proliferation.

Despite P deficiency, respiration rates increased by 25–30% under Pal_500_ treatments (Figure 2C), indicating a metabolic shift toward energy maintenance. This was accompanied by the upregulation of odhA (2-oxoglutarate dehydrogenase) and SAMC1 (S-adenosylmethionine synthase), the genes involved in TCA-cycle reinforcement and methyltransferase activity, respectively [37]. At higher P levels (31.5 mg/L), cells countered Pal toxicity via a slightly enhanced P uptake (Figure 2A), as well as upregulated phosphate transporters in the previous literature [38].

In contrast, Mt treatments had no significant impact on cell density (Figure 2B). Without severe stress, cells did not exhibit compensatory P uptake or metabolic reprogramming, therefore maintaining baseline gene expression (IF2CP, odhA) and growth rates compared with the control [39]. It is worth mentioning that P uptake at high P concentrations (31.5 mg/L) is likely a composite of passive adsorption onto mineral particles’ surfaces and residual cellular uptake by surviving cells.

### 4.3. EPS Composition and Functional Group Dynamics

In our study, cell-surface proteins in EPSs were identified as one of the primary targets of Pal interaction (Figure 4A), a finding consistent with prior studies showing the preferential binding of fibrous clays to polar macromolecules [40]. Unlike Mt, which induced no significant changes in EPS composition, Pal treatment caused a 25–30% reduction in cell-surface protein content and a 10–15% decrease in polysaccharides (Figure 4A,B). This depletion likely reflects the active shedding of EPS components to mitigate Pal’s physical adhesion, as evidenced by the 50% decline in C=O (amide I) and -COOH (carboxylate) groups in the ATR-IR spectra of both Mt and Pal treatments (Figure 5) [14]. These functional groups, critical for electrostatic and hydrogen bonding with Mt and Pal particles, were downregulated as a defensive strategy to disrupt clay mineral attachment.

The 3D-EEM fluorescence spectroscopy further revealed tryptophan-like substances (C1) as the most responsive component to Mt and Pal treatment (Figure 3). The 30% and 18% reduction in C1 intensity under Mt and Pal treatments, respectively, correlates with their ability to complex with aromatic rings in tryptophan via π-π stacking, a mechanism previously observed in clay–organic matter interactions [41]. This preferential binding suggests algae deploy a self-protective EPS remodeling strategy, sacrificing proteinaceous ligands to minimize mineral-induced cellular damage.

### 4.4. Cellular Damage and Adaptive Stress Responses

TEM imaging (Figure 7C,D) revealed that Pal’s fibrous particles penetrated the algal cell wall at concentrations of 500 mg/L, forming protrusions that disrupted the plasma membrane. This structural damage correlated with a 90% increase in soluble protein content (Figure 4A), indicative of intracellular protein efflux from compromised membranes. In response to membrane damage, cells reprogrammed their carbon metabolism by switching the intracellular carbon flux from lipids to polysaccharides to produce protective cell wall matrices [23]. As depicted in Figure 4B, intracellular polysaccharide content increased by 40% under Pal_200_ and 90% under Pal_500_. This adaptive strategy supported survival at sub-lethal Pal concentrations, but 500 mg/L Pal still caused 95% cell death (Figure 7D), overwhelming repair mechanisms. These cumulative effects collectively explain Pal’s severe toxicity to *C. reinhardtii*.

In stark contrast, Mt treatment only induced slight changes in protein or polysaccharide content (Figure 4A,B), suggesting its non-invasive interaction with cells. Unlike Pal, Mt’s layered structure did not penetrate the cell wall or trigger a defensive carbon reallocation, reinforcing its relatively benign impact on algal physiology.

### 4.5. Environmental Implications

Our findings underscore the dual ecological roles of clay minerals in aquatic ecosystems. Fibrous Pal acts as a multi-faceted stressor, imposing both physical barriers to light absorption and a chemical disruption of nutrient uptake, thereby threatening algal fitness and potentially reshaping community dynamics through competitive exclusion [30]. Its ability to induce cellular damage and photosynthetic collapse (Figure 1 and Figure 2) highlights risks for primary productivity in Pal-rich environments.

Conversely, layered Mt functions as a benign biocolloidal carrier, enabling algal-cell adhesion via electrostatic interactions without triggering cytotoxic responses (Figure 4). This property may facilitate beneficial associations in natural systems, such as nutrient retention or biofilm formation, without compromising physiological integrity.

A notable ecological implication is Pal’s induction of DNA leakage from stressed cells (Figure 7D), which could accelerate horizontal gene transfer (HGT) among phytoplankton. By facilitating the spread of stress-adaptive alleles (e.g., membrane repair or detoxification genes), this mechanism may drive rapid evolutionary responses in microbial communities under persistent clay-mineral stress [42]. Such HGT dynamics add a new dimension to understanding mineral–microbe interactions in biogeochemical cycles.

## 5. Conclusions

In this study, significant differences were found in the effects of Mt and Pal on the morphology and physiology of *C. reinhardtii*. Layered Mt attached to the cell surface and could provide positive effects for algal growth. In contrast, fibrous Pal wrapped around the cell surface and significantly inhibited algal photosynthesis. Moreover, some Pal could even insert itself into cells, leading to damage of the cell membrane and eventually cell death. Further research is needed to explore interactions between various microalgae and clay minerals and their influences on the biogeochemical cycles of elements in aqueous environments.

## Data Availability

Data is contained within the article.
